# Using the Multi-Theory Model (MTM) of Health Behavior Change to Explain the Correlates of Mammography Screening among Asian American Women

**DOI:** 10.3390/pharmacy9030126

**Published:** 2021-07-15

**Authors:** Manoj Sharma, Chia-Liang Dai, Kavita Batra, Ching-Chen Chen, Jennifer R. Pharr, Courtney Coughenour, Asma Awan, Hannah Catalano

**Affiliations:** 1 Department of Environmental and Occupational Health, School of Public Health, University of Nevada, Las Vegas, NV 89119, USA; manoj.sharma@unlv.edu (M.S.); Jennifer.pharr@unlv.edu (J.R.P.); courtney.coughenour@unlv.edu (C.C.); 2 Department of Teaching and Learning, College of Education, University of Nevada, Las Vegas, NV 89154, USA; chia-liang.dai@unlv.edu; 3 Office of Research, Kirk Kerkorian School of Medicine, University of Nevada, Las Vegas, NV 89102, USA; 4 Department of Counselor Education, School Psychology, and Human Services, College of Education, University of Nevada, Las Vegas, NV 89154, USA; ching-chen.chen@unlv.edu; 5 Department of Health Informatics, School of Health Sciences, Purdue University, West Lafayette, IN 47906, USA; asmaawan@student.purdueglobal.edu; 6 School of Health and Applied Human Sciences, University of North Carolina Wilmington, Wilmington, NC 28403, USA; catalanoh@uncw.edu

**Keywords:** mammography, multi-level theory, screening, behavior, Asian American

## Abstract

Globally, breast cancer is the most common malignancy affecting women. The incidence of breast cancer has been growing among Asian American women. Mammography is a screening procedure that provides early diagnosis for the timely treatment to reduce premature mortality due to breast cancer. However, there are no national data available that summarize the rates of mammography screening among Asian American women. Some small-scale studies have reported low rates of mammography uptake among Asian American women. This cross-sectional study utilized the fourth-generation, multi-theory model (MTM) of health behavior change to explain the correlates of mammography screening among Asian American women between the ages of 45–54 years. A 44-item instrument was evaluated for face, content, and construct validity (using structural equation modeling) and reliability (Cronbach’s alpha) and administered electronically to a nationally representative sample of Asian American women (*n* = 374). The study found that Asian American women who have had received mammograms in the past 12 months as per recommendations, all three constructs of MTM, namely, participatory dialogue (β = 0.156, *p* < 0.05), behavioral confidence (β = 0.236, *p* < 0.001), and changes in the physical environment (β = 0.426, *p* < 0.001) were statistically significant and crucial in their decision to initiate getting a mammogram, accounting for a substantial 49.9% of the variance in the decision to seek mammography. The study also found that the MTM constructs of emotional transformation (β = 0.437, *p* < 0.001) and practice for change (β = 0.303, *p* < 0.001) were significant for maintaining the repeated behavior of getting annual mammograms and were responsible for 53.9% of the variance. This evidence-based study validates the use of MTM in designing and evaluating mammography screening promotion programs among Asian American women aged 45–54 years.

## 1. Introduction

Globally, cancer is the second leading cause of mortality accounting for approximately 9.6 million deaths [1]. Breast cancer is the most common malignancy affecting women worldwide [2]. In the United States (U.S.), breast cancer is the most commonly diagnosed cancer after skin cancers, with one in eight (13%) women being diagnosed with an invasive type of breast cancer during their lifetime [3]. According to the American Cancer Society’s [4] recent projected estimates, there will be approximately 284,200 new cases and 44,130 deaths attributed to breast cancer in the U.S. in 2021. The differential risk of breast cancer by nativity, racial, and ethnic characteristics was also reported with Asian American women bearing a disproportionate burden [5,6].

The incidence of breast cancer has been growing with a rate of 94 cases per 100,000 among Asian American women residing in the U.S. [5,7]. Previous reports confirmed that the odds of survival were associated with the Asian ethnicity overall [8]. An analysis of this limited SEER registry data from 2019 indicated that there was a total of 47,401 Asian/Pacific Islander women diagnosed with invasive breast cancer between 2012 and 2016 [3]. With regard to the nativity of Asian American women, 132 cases of women with diagnosed breast cancer were compared with 438 Asian American without breast cancer diagnosis [6]. The results of the study suggested that the breast cancer risk is higher among immigrant Asian American women compared with their US-born counterparts or those who have lived less than 50% of their life in the U.S. [6]. The rate of survival also varied across racial and ethnic groups, which highlights the need of detecting breast cancers at an early stage with the help of primary and secondary levels of prevention, including awareness and screening. 

Mammography is a screening procedure that provides early diagnosis, which can lead to treatment and reduced premature mortality due to breast cancer (Centers for Disease Control & Prevention (CDC)) [9]. However, the use of mammograms as a screening method is still a debatable issue due to the problems of false positives, over-diagnosis, and over-treatment [10]. Additionally, health authorities differ in their stances on mammography recommendations across age groups. Four primary authorities in the U.S. proposed guidelines on mammography: American College of Obstetricians and Gynecologists (ACOG), U.S. Preventive Services Task Force, American Cancer Society (ACS), and National Comprehensive Cancer Network (NCCN) [11]. There seems to be some general agreement in these four sets of guidelines that women ages 45–54 years should undergo annual mammograms, and this guideline was used for our study.

To our best knowledge, there are no national data available that summarize the rates of mammography screening among Asian American women through a country-wide population-based survey. However, some small-scale studies have reported low rates of mammography uptake [12]. For instance, a study of Korean American women reported that 22.2% of women ages 45–49 years and 29% of women ages 50–54 years had a mammogram in the past year [12]. According to a 2008 Behavioral Risk Factor Surveillance System (BRFSS) data-based study, 60.3% of Asian American women over the age of 40 years had mammograms in the past year, which was the lowest among all the groups studied [13]. However, the estimates of mammography uptake or utilization are not yet available. Some studies reported regional differences across the U.S. for the mammography screening rates among Asian American women. For instance, a pooled weighted data analysis from five cycles of the California Health Interview Survey conducted between 2001 and 2009, reported an increase in mammograms across all ages in Asian American women from 76–82% from 2001 to 2009 [14]. There is a caveat to this finding; the response rate to this survey was quite low (17.7% to 37.7% in different cycles), which emphasizes the need to conduct additional research pertaining to mammography screening utilization among Asian American women. The Asian American community is increasing rapidly in the U.S. and the present healthcare system and health research are not catering to the needs of this diverse community [13]. In order to meet the growing needs and address the gap in the literature for this underserved minority group, this study is being undertaken [13]. The findings of this study will help in designing behavior change interventions to increase mammography uptake or utilization among Asian American women.

Several factors were known to affect the utilization of mammography screening among Asian American women. Some determinants that are associated with increased rates of mammography screening in Asian American women are having (1) U.S. citizenship [15], (2) longer residency in the U.S. [14,16], (3) a college education [14,17], (4) knowledge of the guidelines [16], (5) health insurance [13,14], (6) a primary care provider who recommended a mammogram [17,18,19], (7) a routine health check-up in the past year [13], and (8) knowing someone with a history of breast cancer and/or having undergone mammography [16]. Some factors that are barriers to getting mammograms among Asian American women are (1) being of Muslim religion [19,20], (2) perceived religious discrimination [19], (3) impingement on modesty [19], (4) being less acculturated [21], and (5) logistical barriers [22]. It is important to note that not all Asian Americans are a monolithic entity and there are variations among the determinants based on national origin. 

There are few interventions that promote mammography among Asian American women. Some of the intervention approaches that have been used are the patient navigator care management model [23], community workshops [24], medical interpretation services for limited English proficiency patients [25], use of primary care providers [26], religiously tailored interventions [27,28], store-based education [29], web-based education delivery [30] among others. Further, very few studies have used behavioral theories as a basis for these interventions. For instance, Boxwala and colleagues [17] and Lee and colleagues [30] used the health belief model, Wu and West [31] used the transtheoretical model, and Sun and colleagues [32] used the prospect theory. Non-theory-based approaches and utilization of older theories do not improve the predictability of the health behavior change. Such approaches lead to doing “same old same old” without advancement of the scientific discipline of health behavior research (HBR). Furthermore, most of the older approaches were about behavior acquisition and newer fourth-generation approaches have been developed that promote behavior “change” instead of mere acquisition and lead to development of precision interventions [33,34]. The first-generation models were about knowledge transfer, the second-generation models were about skill acquisition, third generation models were about behavior acquisition, and the fourth-generation models are about behavior change utilizing constructs from multiple evidence-based theories [33,34]. Therefore, there is a need to utilize recent fourth-generation health behavior change models to explain the correlates of mammography screening and design precise interventions to promote mammography in this understudied and underserved population of Asian American women. 

**Figure 1:**
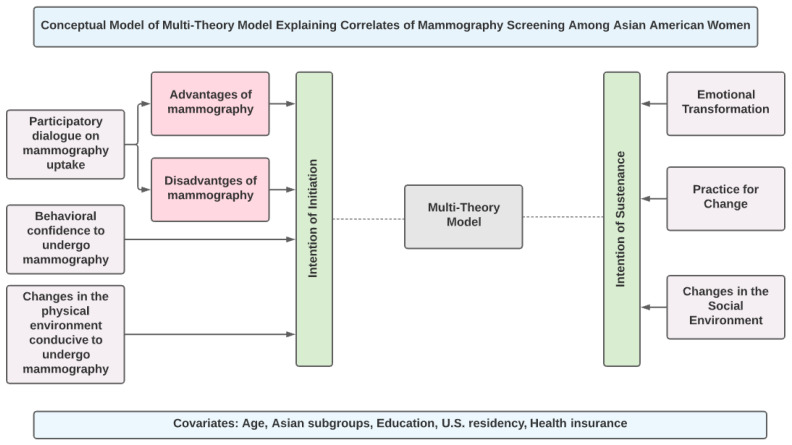
Conceptual model of Multi-Theory Model.

One emerging fourth-generation model is the multi-theory model (MTM) of health behavior change [33,34]. This model breaks down the complex health behavior change into two components of initiation and maintenance with three explanatory constructs for each of the two components (Figure 1). This model has been used in qualitative [35,36], cross-sectional [37,38,39,40], and experimental studies [41,42,43] with a variety of behaviors in different priority populations but has not been applied to understanding mammography screening behaviors. Therefore, the current study aims to utilize the multi-theory model (MTM) of health behavior change to explain the correlates of mammography screening in a sample of Asian American women between the ages of 45–54 years.

## 2. Materials and Methods

### 2.1. Study Design and Data Collection

The data collection for this cross-sectional study was conducted from 10 March 2021 through 18 March 2021, utilizing a research panel of participants facilitated by the market research firm Qualtrics [44]. Qualtrics^®^ (the world’s leading enterprise survey technology solution and XM platform) has been providing samples for over a decade now. Qualtrics partners with over 20 online sample providers to recruit a research panel of participants, which has been created via convenience sampling to build samples from multiple sources. This enable researchers with the diverse and representative datasets. The sample partners randomly select respondents, who are likely to qualify. The majority of the samples come from traditional, actively managed, panel portals. Occasionally, social media is used to gather respondents. For inaccessible groups, Qualtrics utilizes niche panels via special recruitment campaigns [45]. The survey invitations are intentionally kept general to prevent the self-selection bias. Depending upon the study’s inclusion criteria, Qualtrics adds screening questions in the beginning of the survey to ensure inclusion of eligible participants. All qualifying participants who complete the survey are compensated in accordance with Qualtrics^®^ panel agreement. The agreement varies and may include cash, airline miles, charitable donations, sweepstakes entrance, vouchers, gift certificate, etc. Additional information about ethical, methodological, and regulatory guidelines can be found at www.esomar.org (accessed on 8 July 2021). Previous studies have provided the detailed information related to the use of Qualtrics^®^ research panel platforms [45].

### 2.2. Ethical Considerations

The study (protocol #1727672-1, dated 4 March 2021) was considered an exempt research study by the Institutional Review Board (IRB). Participation in the study was completely voluntary, and detailed information about the study’s objectives and significance were provided to participants in an informed consent attached with the web-based survey. Personal identifiers, including name and email address, were not collected to preserve anonymity. Only one response per participant was allowed. We used the “Prevent Ballot Box Stuffing” feature in the Qualtrics to restrict multiple responses from the same participant. This feature is driven by a strong algorithm to ensure data integrity and unique responses. Additionally, Qualtrics utilizes digital fingerprinting technology to preserve integrity of the survey.

### 2.3. Data Intergrity

Data for this study were collected by Qualtrics Research Services as a part of the contractual agreement. As an essential part of the contract, all data privacy laws and regulations were followed to preserve data integrity. Qualtrics database does not hold confidential information of the respondents or panelists. Qualtrics^®^ provided deidentified data to the researchers in an excel sheet, which was stored on a password protected desktop computer in a locked office. Only the principal investigator and statistician of this study had access to the deidentified data files.

### 2.4. Survey Questionnaire 

Based on MTM, a 44-item survey questionnaire was developed to determine the correlates of mammography screening uptake among Asian American women aged 45–54 years. The survey was composed of 13 demographic questions and mammography history, and 31 items for the two primary MTM theoretical constructs (initiation and sustenance). A panel of eight subject matter experts (SMEs), investigated face and content validity of the questionnaire in several rounds of review. The reviews were blinded to prevent observer bias. Several revisions were performed to enhance readability and content of the questionnaire. A detailed description of the instrument’s domains and constructs is provided in Table 1. 

#### 2.4.1. Intention of Initiation 

Constructs of advantages and disadvantages of intention of initiation were measured on a 5-point Likert scale, which ranges from “strongly disagree” to “strongly agree.” 

The score of Participatory dialogue was derived by subtracting the summative score of disadvantages from advantages. The other two constructs of intention to initiation were behavioral confidence and changes in the physical environment. These were also measured on 5-point Likert scale of surety, which had the response options of Not at all sure (0), slightly sure (1), moderately sure (2), very sure (3), completely sure (4). A high score was associated with the likelihood of initiation of behavior change.

#### 2.4.2. Intention of Sustenance

Constructs of emotional transformation, practice for change, and changes in the social environment were measured on a 5-point Likert scale of surety, which has the response options of Not at all sure (0), slightly sure (1), moderately sure (2), very sure (3), completely sure (4). A high score was associated with the likelihood of sustenance of behavior change. Additionally, two items were used to model initiation and sustenance, which were measured as scale variables. 

### 2.5. Statistical Analysis

We used IBM SPSS version 27.0 (IBM Corp. Armonk, NY, USA), Mplus 7.11, and G* Power software packages for the analyses. Minimum sample size was estimated using the Cohen’s effect sizes conventions (based on the type of statistical test) corresponding to 99% power [46,47]. The significance level was set at 0.05, and 95% confidence intervals were reported wherever appropriate. Data normality assumptions were assessed through visual inspection of normal Q-Q plots and histograms. An independent-samples t-test was utilized to compare the mean scores of MTM constructs across groups who have had mammography and those who have not. Categorical variables were expressed as counts and proportions, whereas continuous variables were represented as means and standard deviations. Two separate Hierarchical Regression Models (HRM) were built to predict the variance in the likelihood of initiation and sustenance of mammography behavior by multiple factors, such as demographic characteristics and MTM constructs. 

For construct validation, Structural Equation Modelling (SEM) was utilized to determine the structural relationship between measured variables and latent constructs. Weighted least squares approach (WLSMV estimator) and the 0.05 alpha level were used to test our two hypothesized structural models among the samples. Drawing upon the theory and prior research, our hypothesized model included participatory dialogue (advantages and disadvantages), behavioral confidence, changes in physical environment, changes in social environment, practice for change, and emotional transformation as independent variables; and initiation and sustenance of health behavior change as dependent variables. We hypothesized that (1) the variables of participatory dialogue (advantages and disadvantages), behavioral confidence, and changes in physical environment constructs would impact the initiation of mammography screening behavior change, and (2) the changes in social environment, practice for change, and emotional transformation constructs would impact the sustenance of mammography screening behaviors. This study used a variety of fit indices because they provide different information about model fit. We considered the substantive meaningfulness of the model, significant *χ*^2^ statistics as evidence that models did not fit the data exactly [48], Tucker–Lewis (TLI) and comparative fit (CFI) indices greater than 0.95 evidence of good fit [48,49,50], and root means square error of approximation (RMSEA) values of less than 0.05 to be acceptable [51]. To estimate the sample size for testing hypothesized models, we first sought to meet the conventional 10 subjects per variable ratio [52,53,54]. Next, sample size calculation for Structural Equation Modeling [55] recommended a minimum of 137 participants were required to achieve a statistical power level of 0.8 at an alpha level of 0.05 with 0.3 (medium) anticipated effect size and number of variables in the models. Finally, previous studies suggested that, for confirmatory factor analysis, this sample size was sufficiently powered to evaluate the hypothesized models [56,57,58].

## 3. Results

A total of 374 participants completed this study. The mean age of the study sample was 49.25 (SD: 2.68) years. Chinese Americans represented 41.2% of the sample. The majority of participants (69.3%) were employed, working 34.9 average hours per week. The percentage of participants with health insurance coverage was 93%, over 60% of the population had a household income under $100,000 and lived in suburban neighborhoods. Forty percent of participants reported their religious affiliation as Christianity and 68.7% of the women were married (Table 2). 

There were significant differences in the mean scores for all constructs of initiation and sustenance among those who have had mammography compared with those who have not had mammography. Mammography users had a statistically significant higher mean scores for initiation (3.24 ± 0.90 vs. 1.63 ± 1.2, *p* < 0.001) and sustenance (3.13 ± 1.0 vs. 1.23 ± 1.1, *p* < 0.001) compared to mammography non-users (Table 3).

### 3.1. Asian American Women Following Recommendations on Routine Mammography Screening 

Hierarchical multiple regression was performed to determine if the sequential addition of participatory dialogue, behavioral confidence, and changes in the physical environment improved the likelihood of initiation over the demographic variables of age, Asian subgroups, education, duration of U.S. residency and health insurance (Model 1). The addition of participatory dialogue to the prediction of initiation led to a statistically significant increase in R^2^ of 0.207, F (1, 191) = 12.113, *p* < 0.001 (Model 2). The addition of behavioral confidence to the prediction of initiation led to a statistically significant increase in R^2^ of 0.139, F (1, 190) = 19.198, *p* < 0.001 (Model 3). Among participants following mammography screening recommendations, the full model containing demographic variables and all three constructs to predict initiation was statistically significant, R^2^ = 0.519, F (1, 189) = 25.490, *p* < 0.001; adjusted R^2^ = 0.499 (Model 4). All model results can be found in Table 4.

Hierarchical multiple regression was performed to determine if the sequential addition of emotional transformation and practice for change improved the likelihood of sustenance over the demographic variables of age, Asian subgroups, education, duration of U.S. residency and health insurance (Model 1). In the hierarchical regression with sustenance as the dependent variable, the addition of emotional transformation to the prediction of sustenance led to a statistically significant increase in R^2^ of 0.452, F (1, 191) = 34.119, *p* < 0.001 (Model 2). The addition of practice for change to the prediction of sustenance led to a statistically significant increase in R^2^ of 0.040, F (1, 190) = 34.128, *p* < 0.001 (Model 3). The full model containing preselected demographic variables and three MTM constructs to predict sustenance was statistically significant, R^2^ = 0.558, F (1, 189) = 29.844, *p* < 0.001; adjusted R^2^ = 0.539 (Model 4). All model results can be found in Table 4. 

### 3.2. Asian American Women Not Following Recommendations on Routine Mammography Screening 

Among participants not following mammography screening recommendations, hierarchical multiple regression was performed to determine if the sequential addition of participatory dialogue, behavioral confidence, and changes in the physical environment improved the likelihood of initiation over the demographic variables of age, Asian subgroups, education, duration of U.S. residency and health insurance (Model 1). The addition of participatory dialogue to the prediction of initiation led to a statistically significant increase in R^2^ of 0.202, F (1, 168) = 8.185, *p* < 0.001 (Model 2). The addition of behavior confidence to the prediction of initiation led to a statistically significant increase in R^2^ of 0.090, F (1, 167) = 11.014, *p* < 0.001 (Model 3). The full model containing demographic variables and all three constructs to predict initiation was statistically significant, R^2^ = 0.319, F (1, 166) = 9.709, *p* < 0.001; adjusted R^2^ = 0.286 (Model 4). All model results can be found in Table 5.

Hierarchical multiple regression was performed to determine if the sequential addition of emotional transformation and practice for change improved the likelihood of sustenance over the demographic variables of age, Asian subgroups, education, duration of U.S. residency and health insurance (Model 1). In the hierarchical regression with sustenance as a dependent variable, the addition of emotional transformation led to a statistically significant increase in R^2^ of 0.533, F (1, 168) = 35.205, *p* < 0.001 (Model 2). The addition of practice for change to the prediction of sustenance led to a statistically significant increase in R^2^ of 0.023, F (1, 167) = 32.920, *p* < 0.001 (Model 3). The full model containing demographic variables and three MTM constructs to predict sustenance was statistically significant, R^2^ = 0.594, F (1, 166) = 30.341, *p* < 0.001; adjusted R^2^ = 0.574 (Model 4). All model results can be found in Table 5.

### 3.3. Structural Equation Modelling 

**Figure 2:**
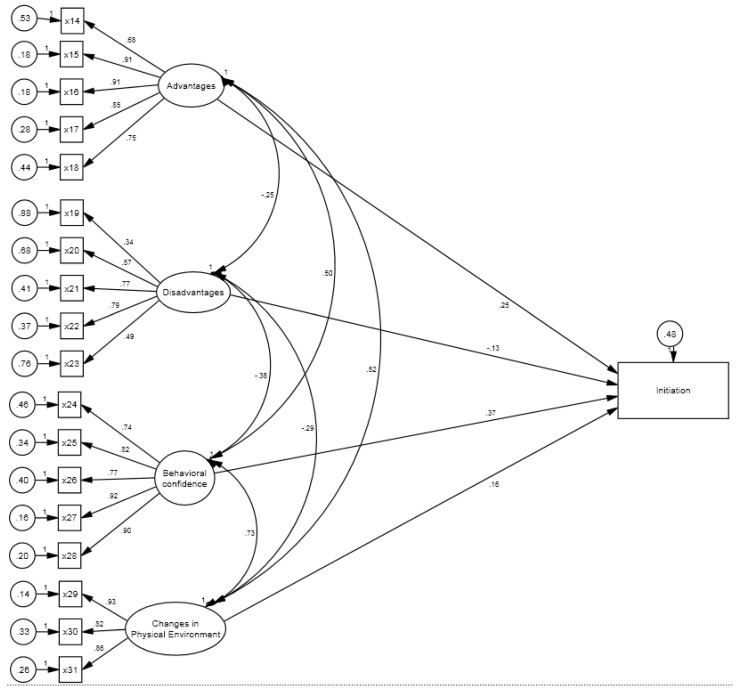
Structural Equation Modeling for Initiation of Mammogram Behavior among Survey Respondents of Asian American women.

We examined two hypothesized models. The results of the initiation model indicated the model fits the data well (e.g., *χ*^2^ [142] = 304.56 [*p* < 0.01], CFI = 0.96, TLI= 0.95, and RMSEA = 0.06) (Table A1). We observed the standardized effects of latent variables on their reflective indicators (i.e., factor loadings) and found an overall pattern of statistically significant loadings for advantages, disadvantages, behavioral confidence, and changes in the physical environment. The advantages had large effects (e.g., β ranging from 0.68 to 0.91) on its five indicators; the disadvantages had moderate effects (e.g., β ranging from 0.34 to 0.79) on its five indicators; the behavioral confidence had large effects (e.g., β ranging from 0.74 to 0.92) on its five indicators; and the changes in the physical environment had large effects (e.g., β ranging from 0.82 to 0.93) on its three indicators (Figure 2, Table A2). These effects suggested that our scale scores provided valid measurement of their constructs. Next, we examined between construct correlations and standardized regression coefficients. We found advantages, behavioral confidence, and changes in the physical environment had small to moderate positive direct effects on the initiation of mammography behavior (e.g., β ranging from.16 to 0.37, *p* < 0.001), while disadvantages had small negative direct effects on the initiation of mammography behavior (β = −0.13, *p* < 0.001). 

**Figure 3:**
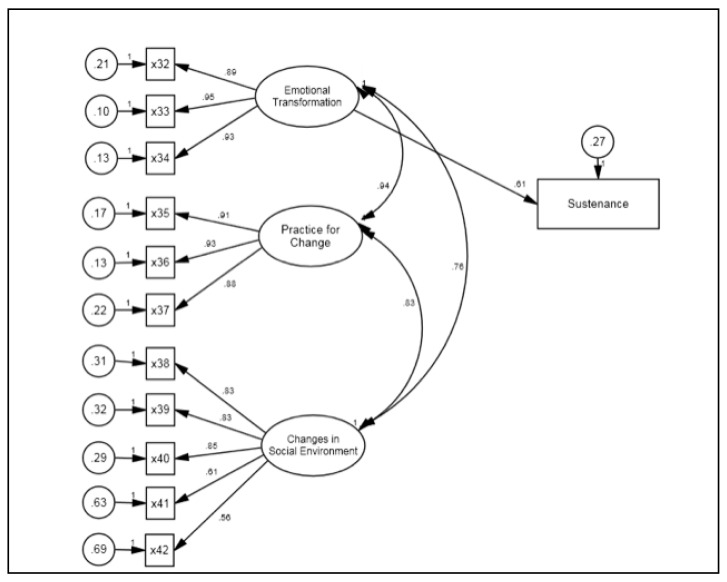
Structural Equation Modeling for Sustenance Model.

For the sustenance model, the fit of the model was excellent (e.g., *χ*^2^ [48] = 152.98 [*p* < 0.01], CFI = 0.97, TLI = 0.95, and RMSEA = 0.08) (Table A1). We observed the standardized effects of latent variables on their reflective indicators (i.e., factor loadings) and found an overall pattern of statistically significant loadings for emotional transformation, practice for change, and changes in the social environment. The emotional transformation had large effects (e.g., β ranging from 0.89 to 0.95) on its three indicators; the practice for change had large effects (e.g., β ranging from 0.88 to 0.93) on its three indicators; and the changes in the social environment had moderate to large effects (e.g., β ranging from 0.56 to 0.85) on its five indicators (Figure 3, Table A3). These effects also suggested that our scale scores provided valid measurement of their constructs. We then examined between construct correlations and standardized regression coefficients for the sustenance model. We found emotional transformation had moderate direct effects on the sustenance of mammography behavior (β = 0.61, *p* < 0.001). However, both practice for change and changes in the social environment did not have any significant effects on the sustenance of mammography behavior.

We found an overall pattern of statistically significant (*p* < 0.05) relationships among the constructs. The negative correlation between disadvantages and other factors was observed (Table 6). The Cronbach’s alphas for all subscales were greater than 0.70 (ranging from 0.72 to 0.95).

## 4. Discussion

The aim of this study was to explain the correlates of mammography screening using the paradigm of MTM in a nationally representative sample of Asian American women aged 45–54 years. The study found that for Asian American women

Who had received mammograms in the past 12 months as per recommendations, all three constructs of the MTM, namely, participatory dialogue (β = 0.156, *p* < 0.05), behavioral confidence (β = 0.236, *p* < 0.001), and changes in the physical environment (β = 0.426, *p* < 0.001) were statistically significant and crucial in their decision to initiate getting the mammogram. In addition, health insurance status (β = 0.127, *p* < 0.05) was also a significant contributor which is also supported by previous literature [13,14]. However, age, Asian subgroups, education, and U.S. residency were not significant contributors in the final model. The final model accounted for a substantial proportion of variance (49.9%) in explaining the decision to receive mammography, which is considered high in social and behavioral sciences [33,34]. While this analysis was not necessary because this group of women were indeed adhering to the guidelines, it was conducted to confirm that the putative MTM constructs are indeed crucial in achieving the starting of the behavior. The study also found that the MTM constructs of emotional transformation (β = 0.437, *p* < 0.001) and practice for change (β = 0.303, *p* < 0.001) were significant for maintaining the repeated behavior of getting mammograms and were responsible for 53.9% of the variance, which again is substantial [33,34]. Similar significant correlates of MTM were observed in the group of Asian American women who had not received a mammogram in the past 12 months, where participatory dialogue (β = 0.294, *p* < 0.001) and behavior confidence (β = 0.310, *p* < 0.001) accounted for 28.6% of the variance in the intention to get mammograms. It is worth noting that the construct of changes in physical environment which was significant for those following mammography recommendations was not significant for those not following the recommendations which is indicative of the barriers that this group may be encountering. These need to be addressed by interventions that promote mammography in Asian American women. Regarding the intention for getting repeat annual mammograms (sustenance), all MTM constructs of sustenance emotional transformation (β = 0.478, *p* < 0.001), practice for change (β = 0.192, *p* < 0.05) changes in the social environment (β = 0.165, *p* < 0.05) were significant contributors accounting for 57.4% of the variance, which is quite considerable [33,34]. It is important to note that all the constructs of MTM are significant in their putative role of explaining continuation of mammography behavior for those Asian American women not following recommendations thus underscoring the relevance of the MTM in designing interventions to promote mammography.

It is worth noting that in this nationally representative sample of Asian American women aged 45–54 years, 46.8% of them had not received a mammogram as per the recommendations of getting one every year [11]. These findings are similar to those from the BRFSS data that found that 39.7% of Asian American women over 40 years had not received mammograms [13]. This is a sizable number of Asian American women who are not following the recommendations, underscoring the need for educational and policy efforts in promoting mammography screening to this subgroup of the population.

On close examination of each construct of MTM in its role associated with mammography screening among Asian American women aged 45–54 years old, we found several important outcomes. First, the participatory dialogue had, as expected, a significantly higher mean score (8.04 ± 5.3) among those who had received a mammogram as per recommendations compared to those who had not (3.86 ± 5.36) (*p* < 0.001). Clearly, those who were convinced of the advantages of getting a mammography screening were motivated to get it. This is also supported by previous studies on determinants of mammography in Asian American women [14,15,16,17,18,19]. However, on hierarchical regression, the construct was found to be significant among those who were adhering to the recommendations but was not significant for those who were not adhering, after adding the construct of changes in the physical environment. This may point to the relative importance of changes in the physical environment construct which has also been supported in the literature in the form of access to health insurance [13,14] or the recommendations from health care providers [17,18,19]. 

The second construct of MTM, behavioral confidence for initiating mammography screening, had the mean score that was significantly higher for those who had mammograms (14.91 ± 3.98) compared with those who had not had mammograms (10.05 ± 4.95) (*p* < 0.001). It was also a significant predictor in the regression models. Previous studies have not examined the extent of the role of this construct, as most of the studies have not utilized behavioral theories for studying determinants of mammography screening among Asian American women [17,18,19]. However, other studies, with other behaviors and other target populations, lend support to this construct of behavioral confidence as playing a significant role in the decision whether to seek mammograms or not [38,39,40]. 

The third MTM construct of changes in the physical environment for initiating the mammography screening had a mean score that was significantly higher for those who had mammograms (10.12 ± 2.23) compared with those who had not had mammograms (8.01 ± 3.09) (*p* < 0.001). It was also a significant predictor in regression models. As discussed earlier, there is evidence from previous research that aspects of the physical environment are very important for Asian American women to seek mammography [13,14,17,18,19]. There is clearly a need to help Asian American women overcome logistical barriers that prevent them from seeking mammography screening [22].

For getting repeated annual mammograms (sustenance), the first MTM construct that was significant was emotional transformation. The mean score for this construct was significantly higher for those who had mammograms (9.42 ± 2.65) compared to those who had not had mammograms (5.02 ± 3.26) (*p* < 0.001). It was also a significant predictor in the regression models. Previous studies have not examined the extent of the role of this construct in improving adherence to mammography screening recommendations among Asian American women because this is a relatively new conceptualization where feelings are purported to be used for goal setting in behavior change [33,34]. However, its role has shown to be important with other behaviors and with other target populations [37,38,39,40] so there is a need to use this construct in planning educational interventions that promote mammography screening among Asian American women.

The mean score on the construct for practice for change, which is another construct that predicts maintenance of behavior, was significantly higher for those who had mammograms (8.85 ± 2.55) compared with those who had not had mammograms (4.82 ± 3.14) (*p* < 0.001). However, on hierarchical regression, the construct was found to be significant among those who were adhering to the recommendations but was not significant for those who were not adhering, after adding the construct of changes in the social environment. This may point to the relative importance of changes in the social environment construct which may play a greater role in the Asian American culture. The role of friends and family members and what they think is an important influence in the lives of Asian Americans. This phenomenon has not been studied extensively in relation to mammography screening among Asian American women, but Somanchi and colleagues [16] found that knowing someone with a history of breast cancer and/or having undergone mammography was a determinant in getting a mammogram for Asian American women. Therefore, this provides support to our conjecture about changes in the social environment playing a greater role. As predictable based on MTM, the mean score of the construct of changes in the social environment was also significantly higher for those who had mammograms (13.16 ± 4.71) compared with those who had not had mammograms (8.69 ± 4.40) (*p* < 0.001). The role of this MTM construct is also supported from research with other behaviors in other target populations [38,39]. There is a need to garner support from social influences in educational programs that promote mammograms among Asian American women.

Since the Asian American community is not a monolithic entity, we collected data on the Asian subgroups. In our sample, the largest representation was from Chinese Americans (41.2%) followed by Filipino Americans (15.8%), followed by South Asian Americans (13.6%) which is more or less representative of the distribution of Asian Americans in the U.S. However, we did not find any significant explanatory potential of these subgroup classifications on predicting potential utilization of mammography screening when MTM constructs are included in the modeling. Future researchers may want to reexamine it more carefully. Previous studies have noted religion, especially being a Muslim, as being a deterrent for getting mammography screening [19,20]. In our sample only nine (2.4%) Asian American women practiced Islam, so we could not analyze this subgroup given the constraints of the small sample size. Future researchers may want to oversample this subgroup to discern if religion indeed is a putative determinant of mammography screening when MTM constructs are taken into consideration.

### 4.1. Implications for Practice

There is a need for both theory-based educational interventions and policy measures that promote mammography screening among Asian American women particularly in the 45–54 age group. The educational interventions can be delivered in primary care settings, OBGYN clinics, community organizations with which Asian American women are associated, faith-based organizations specific to various religions embraced by the Asian American women community, social media, and directed mHealth interventions specifically geared toward this subgroup. MTM can serve as a promising theoretical paradigm in designing and evaluating such interventions. The construct of participatory dialogue in educational interventions can be built by underscoring advantages such as early detection of breast cancer, having peace of mind for self and family, possibility of early treatment, and reduction in premature mortality. Potential barriers such as discomfort, invasion on modesty, inconvenience, and fear of getting a false positive must be discussed and reduced to the extent possible in educational interventions. The construct of behavioral confidence can be fostered through exploration of sources of confidence, using role models, and using stepwise strategies in overcoming barriers. The construct of changes in the physical environment can be mobilized through resources support and reminders. The construct of emotional transformation can be channelized in educational interventions by appealing to the feelings of Asian American women and harnessing these into concrete goals of getting timely mammograms, helping overcome self-doubt, and remaining motivated. The construct of practice for change can be operationalized by encouraging Asian American women to keep records and have reminder systems, overcoming barriers, and making alternate plans if faced with obstacles. Finally, the construct of changes in the social environment must be used by educational programs where family, friends, and healthcare providers should be encouraged to promote, remind, and help with mammography screening. 

### 4.2. Strengths and Limitations 

This study is among the few studies that are based on a behavioral theory to decipher determinants of mammography screening in the high-risk Asian American women community. The study collected data on a nationally representative adequately powered sample representing all subgroups of Asian American women aged 45–54 years. The study utilized a contemporary fourth-generation paradigm of MTM. The psychometric validation of the tool used in the study was done meticulously. However, there were also some limitations to this study. Self-reports were utilized to collect information about mammography. Objective data using medical records could have been used to provide more accuracy. The cross-sectional nature of the design always limits causal inferences because the data on the independent variables (MTM constructs) and dependent variables (intentions) are collected at the same point in time. Future research must look into longitudinal designs. Finally, as mentioned earlier, we did not have adequate representation of the Muslim Asian American women in our sample, so we could not examine the role of MTM constructs while controlling for religion. 

## 5. Conclusions

MTM is a fourth-generation behavioral theory that is gaining popularity and accumulating empirical evidence. This study is among one of those burgeoning studies that provide support to MTM. The Asian American women lag behind their White counterparts in getting screened for breast cancer and availing mammography screening, as verified by this study where almost 47% had not received the recommended mammogram in the past year. MTM-based educational and policy interventions can help Asian American women, particularly those 45–54 years old, meet the recommendations of annual mammograms thereby reducing disparities for this high-risk subgroup in the U.S. population.

## Figures and Tables

**Table 1:** Domains and constructs of MTM theoretical framework.

Domain	Constructs		Definition (s) and Examples from This Study	Number of Items	Possible Range (Min–Max)
Intention of Initiation	Participatory dialogue (Derived after subtracting summative score of disadvantages from advantages)	Advantages	Perception of advantages following the specific behavior initiation, e.g., early detection, peace of mind, etc.	5	0–20 units
		Disadvantages	Perception of disadvantages following the specific behavior initiation, e.g., invasion of modesty, inconvenience, etc.	5	0–20 units
	Behavior confidence		Surety of behavior despite external and internal driving factors, e.g., overcoming cost, overcoming discomfort, etc.	5	0–20 units
	Changes in the physical environment		Overcoming enabling factors for behavior initiation, e.g., easy access to a place, ability to get it when one wants it, etc.	3	0–12 units
Intention of Sustenance	Emotional transformation		Converting emotions into intention, e.g., directing feelings into goal, self-motivation, etc.	3	0–12 units
	Practice for change		Continuous adaptation for behavior changes, e.g., monitoring, overcoming barriers, etc.	3	0–12 units
	Changes in the social environment		Using social cues for behavior change, e.g., support from family, friends, etc.	5	0–20 units

**Table 2:** Demographic characteristics of the sample population (*n* = 374) of Asian American women collected in March 2021.

Variable	*n* (%)
**Years of residency in the U.S.**	
Less than 30 years	166 (44.4)
More than 30 years	208 (55.6)
**Asian sub-groups**	
Chinese American	154 (41.2)
Korean American	22 (5.9)
Filipino American	59 (15.8)
South Asian American	51 (13.6)
Japanese American	40 (10.7)
Others ^1^	36 (9.6)
Prefer not to answer	12 (3.2)
**Religion affiliation**	
Christianity	162 (43.3)
Buddhism	48 (12.8)
Atheist	45 (12)
Hinduism	31 (8.3)
Others including Islam, Judaism and other categories	88 (23.6)
**Residence**	
Rural	28 (7.5)
Urban	110 (29.4)
Suburban	236 (63.1)
**Educational attainment**	
High school graduate or less	15 (4.0)
Some college or trade school	36 (9.6)
Associate’s degree	46 (12.3)
Bachelor’s degree	175 (46.8)
Master’s degree or above	102 (27.3)
**Health insurance status**	
Yes	348 (93)
No	26 (7)
**Household income (USD)**	
<25,000	24 (6.4)
25,000–50,000	65 (17.4)
50,001–75,000	72 (19.3)
75,001–100,000	65 (17.4)
>100,000	148 (39.5)
**Marital status**	
Married	257 (68.7)
Never married	52 (13.9)
Divorced/separated/widowed	51 (13.6)
Others ^2^	14 (3.8)
**Employment status**	
Yes	259 (69.3)
No	115 (30.7)

^1^. Others Asian subgroups include Vietnamese American, Middle East American, and other South East Asian American.; ^2^. Others in marital status include those in a civil union or registered domestic partnership and a member of an unmarried couple.

**Table 3:** Comparing mean scores of multi-theory model constructs of behavior change across groups of survey respondents of Asian American women.

Groups	Women Who Have Had Mammography (*n* = 199)			Women Who Have Not Had Mammography (*n* = 175)			
Constructs	Possible Score Range	Observed Score Range	Mean ± SD	Possible Score Range	Observed Score Range	Mean ± SD	*p*-Value *
Initiation	0–4	0–4	3.24 ± 0.90	0–4	0–4	1.63 ± 1.2	<0.001
Participatory dialogue: advantages	0–20	4–20	17.11 ± 2.96	0–20	2–20	14.48 ± 3.7	<0.001
Participatory dialogue: disadvantages	0–20	0–20	9.07 ± 3.78	0–20	0–20	10.62 ± 3.46	<0.001
Participatory dialogue **	−20–[+20]	−13–[+20]	8.04 ± 5.3	−20–[+20]	−12–[+20]	3.86 ± 5.36	<0.001
Behavior confidence	0–20	1–20	14.91 ± 3.98	0–20	0–20	10.05 ± 4.95	<0.001
Changes in the physical environment	0–12	2–12	10.12 ± 2.23	0–12	0–12	8.01 ± 3.09	<0.001
Sustenance	0–4	0–4	3.13 ± 1.0	0–4	0–4	1.23 ± 1.1	<0.001
Emotional transformation	0–12	0–12	9.42 ± 2.65	0–12	0–12	5.02 ± 3.26	<0.001
Practice for change	0–12	1–12	8.85 ± 2.55	0–12	0–12	4.82 ± 3.14	<0.001
Changes in the social environment	0–20	0–20	13.16 ± 4.71	0–20	0–20	8.69 ± 4.40	<0.001

* *p* values less than 0.05 are considered statistically significant; ** Participatory dialogue is computed by subtracting disadvantages from advantages.

**Table 4:** Hierarchical Multiple Regression (HRM) predicting likelihood for initiation and sustenance of mammogram behavior among survey respondents of Asian American women following recommendations on routine mammography screening (*n* = 199).

Variables	Model 1		Model 2		Model 3		Model 4	
	B	*β*	B	*β*	B	*β*	B	*β*
**The Likelihood for initiation as a dependent variable**								
Constant	1.936		0.281		−0.099		−1.042	
Age	0.005	0.015	0.020	0.065	0.013	0.043	0.019	0.063
Asian subgroups	−0.008	−0.021	−0.020	−0.054	−0.012	−0.034	−0.012	−0.032
Education	−0.041	−0.071	−0.006	−0.011	−0.007	−0.012	−0.016	−0.028
U.S. Residency	0.011 **	0.188	0.009 *	0.157	0.003	0.055	0.001	0.024
Health Insurance	1.008 *	0.163	1.195 **	0.194	0.921 **	0.149	0.784 *	0.127
Participatory dialogue			0.076 **	0.465	0.043 **	0.261	0.026 *	0.156
Behavioral confidence					0.096 **	0.439	0.051 **	0.236
Changes in the physical environment							0.166 **	0.426
R^2^	0.069		0.276		0.414		0.519	
F	2.840 *		12.113 **		19.198 **		25.490 **	
Δ R^2^	0.069		0.207		0.139		0.105	
Δ F	2.840 *		54.521 **		44.971 **		41.145 **	
**The Likelihood for sustenance as a dependent variable**								
Constant	0.937		−0.210		−0.277		−0.368	
Age	0.024	0.069	0.014	0.039	0.012	0.033	0.013	0.037
Asian subgroups	−0.004	−0.010	−0.021	−0.051	−0.019	−0.046	−0.020	−0.047
Education	−0.010	−0.016	0.012	0.019	0.008	0.011	0.008	0.013
U.S. Residency	0.015 **	0.221	0.001	0.016	0.002	0.025	0.002	0.025
Health Insurance	0.578	0.082	0.111	0.016	0.080	0.011	0.103	0.014
Emotional transformation			0.267 **	0.707	0.168 **	0.447	0.164 **	0.437
Practice for change					0.129 **	0.327	0.119 **	0.303
Changes in the social environment							0.010	0.047
R^2^	0.066		0.517		0.557		0.558	
F	2.694 *		34.119 **		34.128 **		29.844 **	
Δ R^2^	0.066		0.452		0.040		0.001	
Δ F	2.694 *		178.773 **		17.014 **		0.493	

B (Unstandardized coefficient); β (Standardized coefficient), * *p*-value < 0.05; ** *p*-value < 0.001; Adjusted R^2^ of initiation = 0.499; Adjusted R^2^ of sustenance = 0.539.

**Table 5:** Hierarchical Multiple Regression (HRM) predicting likelihood for initiation and sustenance of mammogram behavior among survey respondents of Asian American women not following recommendations on routine mammography screening (*n* = 175).

Variables	Model 1		Model 2		Model 3		Model 4	
	B	*β*	B	*β*	B	*β*	B	*β*
**The Likelihood for initiation as a dependent variable**								
Constant	1.679		1.298		1.196		1.028	
Age	−0.013	−0.029	−0.015	−0.033	−0.020	−0.043	−0.018	−0.039
Asian subgroups	0.030	0.067	0.042	0.096	0.056	0.126	0.054	0.121
Education	0.031	0.040	0.045	0.058	0.037	0.047	0.031	0.039
U.S. Residency	−0.002	−0.025	0.000	0.004	−0.007	−0.081	−0.007	−0.086
Health Insurance	0.444	0.127	0.318	0.091	0.161	0.046	0.173	0.050
Participatory dialogue			0.098 **	0.452	0.066 **	0.304	0.064 **	0.294
Behavioral confidence					0.082 **	0.350	0.073 **	0.310
Changes in the physical environment							0.026	0.071
R^2^	0.025		0.226		0.316		0.319	
F	0.852		8.185 **		11.014 **		9.709 **	
Δ R^2^	0.025		0.202		0.090		0.003	
Δ F	0.852		43.772 **		21.885 **		0.704	
**The Likelihood for sustenance as a dependent variable**								
Constant	0.608		−1.312		−1.418		−1.205	
Age	0.013	0.027	0.026	0.055	0.028	0.060	0.022	0.047
Asian subgroups	0.041	0.091	0.035	0.078	0.030	0.066	0.024	0.054
Education	−0.032	−0.040	0.013	0.016	0.003	0.004	−0.006	−0.007
U.S. Residency	−0.006	−0.078	−0.016 **	−0.192	−0.016 **	−0.199	−0.166 **	−0.188
Health Insurance	0.265	0.074	0.268	0.075	0.276	0.077	0.213	0.060
Emotional transformation			0.270 **	0.742	0.184 **	0.507	0.174 **	0.478
Practice for change					0.106 **	0.280	0.072 *	0.192
Changes in the social environment							0.044 *	0.165
R^2^	0.024		0.557		0.580		0.594	
F	0.847		35.205 **		32.920 **		30.341 **	
Δ R^2^	0.024		0.533		0.023		0.014	
Δ F	0.847		201.956 **		9.067 **		5.744 *	

B (Unstandardized coefficient); β (Standardized coefficient), * *p*-value < 0.05; ** *p*-value < 0.001; Adjusted R^2^ of initiation = 0.286; Adjusted R^2^ of sustenance = 0.574.

**Table 6:** Summary of bivariate correlations, means, standard deviations, and internal consistency estimates for study variables using data from Asian American women.

Variables	1	2	3	4	5	6	7
1. Advantages	-						
2. Disadvantages	−0.23 *	-					
3. Behavioral Confidence	0.46 *	−0.37 *	-				
4. Physical Environment	0.48 *	−0.28 *	0.68 *	-			
5. Emotional Transformation	0.52 *	−0.32 *	0.72 *	0.61 *	-		
6. Practice for Change	0.53 *	−0.32 *	0.75 *	0.64 *	0.88 *	-	
7. Changes in Social Environment	0.53 *	−0.30 *	0.65 *	0.64 *	0.69 *	0.74 *	-
*M*	15.88	9.79	12.64	9.13	7.36	6.97	11.07
*SD*	3.57	3.71	5.08	2.87	3.68	3.48	5.08
*α*	0.91	0.72	0.92	0.90	0.95	0.93	0.87

Note. *n* = 374, * *p* < 0.05.

## Data Availability

The data presented in this study are available on request from the corresponding author. The data are not publicly available due to ethical reasons.

## Appendix A

**Table A1:** Values of Selected Fit Statistics for Hypothesized Measurement and Full Structural Models.

Hypothesis/Model	*χ* ^2^	*df*	RMSEA (90% CI)	CFI	TLI
Initiation model	304.56 **	142	0.06(0.05–0.06)	0.96	0.95
Sustenance Model	152.98 **	48	0.08(0.06–0.09)	0.97	0.95

** *p* < 0.01.

**Table A2:** Unstandardized and Standardized Parameter Estimates of Initiation Model.

			*b*	*SE*	*p* *	*β*
Initiation Model						
x14	←	Advantages	1.000	-	-	0.682
x15	←	Advantages	1.326	0.122	<0.001	0.907
x16	←	Advantages	1.310	0.118	<0.001	0.907
x17	←	Advantages	1.319	0.127	<0.001	0.847
x18	←	Advantages	1.221	0.115	<0.001	0.750
x19	←	Disadvantages	1.000	-	-	0.344
x20	←	Disadvantages	1.623	0.301	<0.001	0.565
x21	←	Disadvantages	2.292	0.393	<0.001	0.766
x22	←	Disadvantages	2.284	0.399	<0.001	0.793
x23	←	Disadvantages	1.212	0.234	<0.001	0.486
x24	←	Behavioral Confidence	1.000	-	-	0.737
x25	←	Behavioral Confidence	1.072	0.065	<0.001	0.815
x26	←	Behavioral Confidence	1.060	0.065	<0.001	0.773
x27	←	Behavioral Confidence	1.180	0.071	<0.001	0.915
x28	←	Behavioral Confidence	1.150	0.065	<0.001	0.895
x29	←	Physical Environment	1.000	-	-	0.925
x30	←	Physical Environment	0.965	0.048	<0.001	0.818
x31	←	Physical Environment	1.014	0.047	<0.001	0.862
Initiation	←	Advantages	0.594	0.135	<0.001	0.249
Initiation	←	Disadvantages	−0.446	0.169	<0.001	−0.130
Initiation	←	Behavioral Confidence	0.545	0.113	<0.001	0.371
Initiation	←	Physical Environment	0.223	0.102	<0.001	0.156
Advantages		Disadvantages	−0.052	0.019	<0.001	−0.248
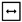
Behavioral Confidence		Advantages	0.243	0.033	<0.001	0.500
Behavioral Confidence		Disadvantages	−0.128	0.036	<0.001	−0.380
Physical Environment		Advantages	0.257	0.032	<0.001	0.515
Physical Environment		Disadvantages	−0.099	0.031	<0.001	−0.286
Physical Environment		Behavioral Confidence	0.592	0.060	<0.001	0.730

* Marker variable *p* values are from tests of standardized effect significance. ← represents connections between nodes (variables).  indicates covariance of residuals between sets of variables.

**Table A3:** Unstandardized and Standardized Parameter Estimates for Sustenance Model.

			*b*	*SE*	*p* *	*β*
Sustenance Model						
x32	←	Emotional Transformation	1.000	-	-	0.888
x33	←	Emotional Transformation	1.126	0.032	<0.001	0.947
x34	←	Emotional Transformation	1.045	0.035	<0.001	0.931
x35	←	Practice for Change	1.000	-	-	0.909
x36	←	Practice for Change	1.007	0.035	<0.001	0.932
x37	←	Practice for Change	0.935	0.042	<0.001	0.884
x38	←	Social Environment	1.000	-	-	0.831
x39	←	Social Environment	1.150	0.061	<0.001	0.827
x40	←	Social Environment	1.048	0.071	<0.001	0.845
x41	←	Social Environment	0.897	0.082	<0.001	0.610
x42	←	Social Environment	0.840	0.084	<0.001	0.557
Sustenance	←	Emotional Transformation	0.785	0.173	<0.001	0.612
Emotional Transformation		Practice for Change	1.204	0.087	<0.001	0.937
Emotional Transformation		Social Environment	0.784	0.073	<0.001	0.761
Practice for Change		Social Environment	0.863	0.074	<0.001	0.826

* Marker variable *p* values are from tests of standardized effect significance. ← represents connections between nodes (variables).  indicates covariance of residuals between sets of variables.
